# Post Stroke Seizures and Epilepsy: From Proteases to Maladaptive Plasticity

**DOI:** 10.3389/fncel.2019.00397

**Published:** 2019-09-13

**Authors:** Keren Altman, Efrat Shavit-Stein, Nicola Maggio

**Affiliations:** ^1^Department of Neurology, The Chaim Sheba Medical Center, Ramat Gan, Israel; ^2^Sackler Faculty of Medicine and Sagol School of Neuroscience, Tel Aviv University, Tel Aviv-Yafo, Israel; ^3^Talpiot Medical Leadership Program, The Chaim Sheba Medical Center, Ramat Gan, Israel

**Keywords:** thrombin, seizure, post stroke epilepsy, proteases-activated receptor 1, ischemic stroke

## Abstract

Post stroke epilepsy (PSE) is the most common cause of seizures in the elderly, yet its underlying mechanism is poorly understood. The classification of PSE is confusing, and there is neither a clear agreement on its incidence and prognosis nor a consensus about specific treatments. The diagnosis of PSE requires the occurrence of late seizures: epileptic events occurring 1 week or more after an ischemic stroke. Late seizures differ from early seizures by the presence of permanent structural changes in the brain. Those structural changes cause a shift in the regulation of neuronal firing and lead to circuit dysfunctions, and thus to a long-term epileptic condition. The coagulation cascade and some of its major components, serine proteases such as thrombin, are known to participate in the acute phase of a stroke. Recent discoveries found that thrombin and its protease-activated receptor 1 (PAR1), are involved in the development of maladaptive plasticity. Therefore, we suggest that thrombin and PAR1 may have a role in the development of PSE by inducing permanent structural changes after the ischemic events toward the development of epileptic focuses. We are confident that future studies will lead to a better understanding of the pathophysiology of PSE, as well as development of more directed therapies for its treatment.

## Introduction

Cerebrovascular diseases are a major cause of disability worldwide and are the most common cause of seizure in the elderly population (Tomari et al., 2017) As stroke contributes to a significant portion of newly diagnosed epilepsy cases (Beghi and Giussani, 2018; Feyissa et al., 2019), there is an urgent need to further investigate this co-morbidity, which is yet poorly understood. A major cause for this lack of information lies in the high variance of the incidence of stroke-related seizures which range from 2 to 20% among different studies (Wang et al., 2017). The lack of clear epidemiological data both alters the ability to assess the impact and significance of post stroke epilepsy (PSE) as well as humpers clinical care by leaving many cases misdiagnosed and untreated. The major causes for this variability derive from multiple stroke etiologies (Stefanidou et al., 2017), inconsistent definitions of stroke related seizure and PSE (Stefanidou et al., 2017; Zhao et al., 2018), variations in time of follow up (Stefanidou et al., 2017; Zhao et al., 2018) and lack of organized protocols (Pitkänen et al., 2016).

As a consequence of these discrepancies, little is known about the relatively common condition of stroke related seizure. A limited comprehension of the mechanisms underlying the connection between stroke and epilepsy are significant as it contributes to the faults described in clinical evaluation and treatment. Fortunately, recent discoveries involving activation of the coagulation cascade in the context of blood brain barrier (BBB) breakdown after stroke are providing novel data on the possible pathophysiological mechanisms of PSE which may lead to novel diagnostic and therapeutic tools. In this review, we will shortly describe the state-of-the-art knowledge on early seizure (ES) and PSE, discuss the role of the proteases involved in this process and try to elucidate how proteases are related to development of circuit dysfunction and maladaptive plasticity leading to epilepsy in the context of ischemic stroke.

## Post Stroke Seizures and Epilepsy

In an attempt to settle the discrepancies mentioned above, *The International League Against Epilepsy* defined criteria that try to differentiate between two types of stroke related seizure by their time of onset. ES occurs within a week after stroke and a late seizure (LS) takes place at least a week or more after an ischemic event (Arboix et al., 2003; Lamy et al., 2003; Xu, 2019). This separation has a critical and functional importance: the pathological mechanisms underlying both conditions seem to have distinct differences. ES is related to acute ischemic changes such as hypoperfusion, variations in calcium and sodium concentrations and glutamate release (Myint, 2006). This temporary ischemia may lead to uncontrolled epileptic activity that halts when the patient passes the acute phase. Differently, LS is a long-term continuing disorder resulting from the permanent structural and functional remodeling of damaged brain areas after an ischemic stroke. Importantly, unlike ES, this long term condition has high probability to lead to permanent changes in neuronal excitability (Silverman et al., 2002), an hyperexcitability state leading to increased risk of epileptic activity. PSE diagnosis is based on the presence of two recurrent seizures, which were not provoked by any factor (metabolic, toxic or other) and did not occur in the acute phase of stroke (Sarecka-Hujar and Kopyta, 2019). Therefore, PSE diagnosis does not follow ES, only the occurrence of a LS is required (Zhao et al., 2018).

It is hard to predict which patient will develop ES, LS, and PSE, but certain risk factors are involved. For example, specific stroke types, such as hemorrhagic stroke and total anterior circulation stroke are strongly correlated with increased chance of PSE (Leone et al., 2009; Graham et al., 2013). ES, with an overall prevalence of 3.8% (Feher et al., 2019), is also correlated with similar stroke groups, being more common after hemorrhagic (8.4%) compared to ischemic stroke (2.4%) (Szaflarski et al., 2008). The extent of cortical injury is also considered to be an important issue for both ES and LS, as studies have shown that the involvement of the parietotemporal cortex, supramarginal gyrus and superior temporal gyrus seems to be connected to post stroke epileptogenesis (Zhao et al., 2018). Lacunar strokes are also associated with PSE, accounting for 11% of PSE cases (Pitkänen et al., 2016; Zhao et al., 2018). However, they are less representative than ischemic events with significant cortical involvement (Pitkänen et al., 2016).

Management of ES and PSE is of major importance, yet the present guidelines do not give a definitive curriculum for the management of those patients. While the current criteria for antiepileptic drug selection are based on the specific individual background of the patient, no consideration on either seizures pathogenesis or risk factors to develop ES and PSE in the future (De Reuck, 2009; Gilad, 2012) are part of the clinical decision process. The identification of the risk for developing ES and PSE through better classification may allow precise treatment that will benefit patients that are currently managed as generic focal epilepsy with no regard to prior cerebrovascular event (Wang et al., 2017). According to recent studies, nearly 35% of the patients with ES, LS, and PSE are resistant to antiepileptic treatment. Critically, both the drug resistant and seizure free patients suffer more of drugs adverse effects than general epilepsy patients (Xu, 2019). Additional limitations can be found regarding the prediction of PSE patient outcome. Different studies have shown either an increased (Arntz et al., 2015) or no association (Arntz et al., 2013) of PSE to risk of mortality or disability based on Modified Rankin Scale. If in the future better diagnostic and prognostic tools will be found, medical decisions regarding PSE management may be dealt in a more precise manner then it is today.

## Proteins and Proteases Involved in ES and PSE

In recent years, several studies have suggested a novel approach which may be key for understanding the basic pathological mechanisms associated with the development of ES and PSE. This novel approach is targeting a number of key proteins and proteases involved in the acute phase of stroke that can increase the risk to develop epileptic conditions (Lee et al., 1997; Isaeva et al., 2012; Terunuma et al., 2019). Thrombin, a serine protease derived from the prothrombin cleavage by activated factor X, has a major role in the coagulation cascade (Coughlin, 2000; Göbel et al., 2018) since it mediates the conversion of fibrinogen to fibrin and activates other coagulation factors V, VIII, XI, XIII, and protein C. Thrombin signaling depends on binding to its receptor, the protease-activated receptor (PAR1), a member of the G coupled receptor family, and has a major influence on endothelial disruption, cytotoxicity and inflammation (Coughlin, 2000; Chen et al., 2010; Pleşeru and Mihailă, 2018). Thrombin also serves as an important therapeutic target. Dabigatran, a non-vitamin K antagonist oral anticoagulant approved for stroke prevention in atrial fibrillation patients, is a direct thrombin inhibitor (Alberts et al., 2012).

**FIGURE 1:**
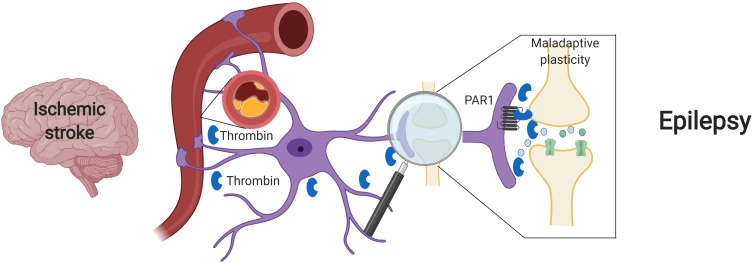
Suggested model – Ischemic stroke leading to maladaptive plasticity and PSE: Following a cerebral vascular occlusion, thrombin increases in the brain. This increase may be as a result of BBB breakdown and entrance of vary blood components into the brain parenchyma or by brain tissue intrinsic production. Thrombin and its main receptor PAR1 enhance NMDA receptor activity and calcium entry, thus leading to a hyperexcitable state and to maladaptive plasticity. Eventually, synchronized epileptic activity occurs as a part of a PSE condition. Created with BioRender.

Beyond its major role in cerebrovascular disease, thrombin has also been pointed as main character in the development of ES after stroke. In acute stroke, cytokine activity promotes an intense neuroinflammation, which underlies BBB disruption. Loss of BBB integrity induces an increase in permeability, facilitating influx of blood components into the brain parenchyma (Abdullahi et al., 2018; Yang et al., 2019). Among various blood components, there is also penetration of serine proteases, such as plasmin and thrombin (Gingrich and Traynelis, 2000), that in turn activates PAR receptors (Ben Shimon et al., 2015). This activation enhances NMDA receptor and calcium overload, thus inducing glutamate mediated neurotoxicity (Gingrich et al., 2000; Stein et al., 2015). Excitatory and inhibitory synapses may be both involved in this process, either through Bestrophin-1 anion channel opening and consequent PAR1 activation in astrocytes (Park et al., 2015) and/or a direct activation of PAR1 in inhibitory interneurons (Maggio et al., 2013). In the former case, through an astrocytic mediated response, an increase in synchronous neuronal firing is achieved (De Pittà and Brunel, 2016; Murphy et al., 2017). In the latter scenario, the activation of PAR1 in interneurons might directly affect IPSCs and thus enhance the excitability of the neuronal network (Maggio et al., 2013). In both cases, a significant hyperexcitable state is established and contributes to seizure onset in the acute phase of stroke (Figure 1).

Thrombin and PAR1 signaling contribution to ES is relatively clear. However, knowledge about their action in late cortical remodeling remains limited. A possible late outcome of their signaling could modify physiological plasticity and eventually lead to the development of maladaptive plasticity that can trigger PSE. Future research should seek to unravel how and when this critical shift occurs.

## Stroke as a Leading Cause for Maladaptive Plasticity and Circuit Dysfunction

One of the main phenomena involved in synaptic plasticity is long term potentiation (LTP), a process which induces active strengthening of synapses, thus posing an important part of memory and learning processes (Rumpel, 2005; Nabavi et al., 2014). LTP requires the activation of NMDA receptors (NMDAR), which in turn leads to increased calcium influx, a hallmark of synaptic plasticity. The calcium influx induces the expression of more NMDAR as well as increasing axonal sprouting and the formation of new synapses, further strengthening the synapse in the process of positive feedback (Zhou et al., 2015; He and Jin, 2016). LTP is input specific, since it is originated and spread only through relevant synapses that will participate in plasticity process (Luscher and Malenka, 2012).

Proteins and proteases involved in ES and PSE pathophysiology also contribute to homeostatic plasticity. Under normal conditions, PAR1 has several functions in the CNS, such as mediating nerve growth factor release and astrocyte proliferation (Noorbakhsh et al., 2003). In the hippocampus, the major expression of PAR1 is in astrocytes, which are responsible for glutamate release and NMDAR enhancement, crucial for LTP process (Almonte et al., 2013). Additionally, it has been shown that thrombin effects, via PAR1 activation, are dose dependent. Low doses of thrombin enhances plasticity, contributing to learning and memory formation, while high concentration, which typically occurs after stroke, inhibit this physiological pathway and activate a pathological form of plasticity (Ben Shimon et al., 2015).

Under stress, such as the one caused by oxygen and glucose deprivation, the role of ischemic LTP (iLTP), a pathological form of LTP, alters physiological plasticity.

Ischemic LTP and LTP have shared mechanisms, since both work through activation of NMDAR, but also bear major differences. iLTP is not input specific like physiological LTP, since it is more specific to NMDA and not necessarily linked to memory (Lenz et al., 2015). Additionally, it has been shown that iLTP can inhibit physiological LTP (Lyubkin et al., 1997), resulting in deficits of hippocampal LTP thus leading to cognitive deficits (Orfila et al., 2018). Therefore, iLTP can enhance a process called maladaptive plasticity, which leads to a restructure of neuronal network and consequently disruption of function (Ben Shimon et al., 2015).

Under acute ischemic conditions, an excessive glutamate influx drives toxic processes such as extreme excitotoxicity and hyperexcitability (Lai et al., 2014). To protect the brain and compensate for neuronal loss, plasticity processes occur through mechanisms such as iLTP (Stein et al., 2015). However, as mentioned above, iLTP can also lead to pathological forms of plasticity, or maladaptive plasticity and consequently a loss of function. This process can lead eventually to the formation of synchronized neuronal circuits, that in turn can end up as epileptic focuses (Cerasa et al., 2014). Interestingly, it has been shown that, under normal conditions, PAR1 inhibition has no influence on dendritic morphology, which is affected during iLTP process. This implicates that PAR1 targeted therapy could be effective under iLTP conditions, without altering normal brain tissue (Schuldt et al., 2016). While iLTP processes occur in the early phase of ischemic stroke and lead to ES, the mechanism leading to PSE remains unclear. Accumulating evidence suggests that BBB damage and exposure to blood protein and proteases may be involved also in LS and epilepsy (Figure 1). For example, recent studies point that albumin plays a role in epileptogenesis after BBB breakdown. In this context, albumin, through TGF-β signaling activation in astrocytes, leads to abnormal reorganization of neuronal circuits and excitatory synaptogenesis, facilitating seizure. Similar mechanisms could also play a role in PSE, as a synergic interaction of proteins and proteases such as thrombin can result in long-term maladaptive plasticity (Friedman et al., 2009; Kim et al., 2017).

Another consequence of maladaptive plasticity is linked to the cognitive outcome. Stroke is known as a major cause of long term cognitive decline (Cao et al., 2015). Additionally, recent studies have shown that thrombin induced neurotoxicity also plays a role in neurodegenerative processes and leads to cognitive deficits (Mhatre, 2004; Stein et al., 2015) which involve damage to reference memory and latency (Mhatre, 2004). Specific analysis of thrombin toxicity in ischemic stroke animal models has also identified damage to similar cognitive functions that could be rescued using direct thrombin inhibitors (Chen et al., 2012).

The role of the serine proteases in the late phase of ischemia and their contribution to maladaptive plasticity and PSE development remains still unclear. A better comprehension of the influence of brain thrombin in the development of seizures is required to gain deeper understanding of these pathological processes for the development of targeted therapy.

## Summary and Conclusion

Post stroke epilepsy is a significant cause of epilepsy, yet the underlying mechanisms leading to this condition are mostly unknown. Better understanding of PSE pathology and its connection to ES is a crucial step forward, since it is possible that proper management and treatment of ES might modify the processes of maladaptive plasticity and delay or prevent PSE development. The lack of precise therapies will continue to be a major obstacle going forward, as yet no clinical trials have been set to compare the effectiveness of existing antiepileptic drugs in PSE management.

Additionally, emerging mechanisms found in recent years might provide a possibility to repurpose drugs indicated to other pathologies such as ischemic stroke to ES and PSE patients. In acute stroke, as a result of thrombin enhancement and iLTP induction, the strengthening of excitatory synapses leads to an hyperexcitable state and facilitates ES. Therefore, it can be beneficial to asses in future studies whether treatment of stroke patients with anticoagulants, specifically thrombin inhibitors, might prevent the onset of maladaptive plasticity and possibly reduce the incidence of PSE. If a significant difference is found, it may be beneficial to also study the impact of anticoagulation therapy on seizure frequency among other populations of epileptic patients. Surely, careful selection of patients is needed to test this latter hypothesis: patients with generalized tonic clonic seizures may be at high risk for hemorrhages in case of falls.

All in all, as the incidence of PSE is expected to increase in the incoming decade, more research toward the understanding of PSE mechanisms is urgently needed. A possible search for better PSE animal models as well as the seek of possible biomarkers (i.e., levels of in thrombin CSF) will improve patient management in the future.

## Author Contributions

All authors conceived and wrote the manuscript.

## Conflict of Interest Statement

The authors declare that the research was conducted in the absence of any commercial or financial relationships that could be construed as a potential conflict of interest.

## References

[B1] Abdullahi W. Tripathi D. Ronaldson P. T. (2018). Blood-brain barrier dysfunction in ischemic stroke: targeting tight junctions and transporters for vascular protection. *Am. J. Physiol. Physiol.* 315 C343–C356. 10.1152/ajpcell.00095.2018 29949404 PMC6171039

[B2] Alberts M. J. Bernstein R. A. Naccarelli G. V. Garcia D. A. (2012). Using dabigatran in patients with stroke. *Stroke* 43 271–279. 10.1161/STROKEAHA.111.622498 22156688

[B3] Almonte A. G. Qadri L. H. Sultan F. A. Watson J. A. Mount D. J. Rumbaugh G. (2013). Protease-activated receptor-1 modulates hippocampal memory formation and synaptic plasticity. *J. Neurochem.* 124 109–122. 10.1111/jnc.12075 23113835 PMC3518655

[B4] Arboix A. Comes E. García-Eroles L. Massons J. B. Oliveres M. Balcells M. (2003). Prognostic value of very early seizures for In-Hospital mortality in atherothrombotic infarction. *Eur. Neurol*. 50 78–84. 10.1159/000072503 12944711

[B5] Arntz R. M. Maaijwee N. A. M. Rutten-Jacobs L. C. A. Schoonderwaldt H. C. Dorresteijn L. D. van Dijk E. J. (2013). Epilepsy after TIA or stroke in young patients impairs long-term functional outcome: the FUTURE Study. *Neurology* 81 1907–1913. 10.1212/01.wnl.0000436619.25532.f3 24174587

[B6] Arntz R. M. Rutten-Jacobs L. C. A. Maaijwee N. A. M. Schoonderwaldt H. C. Dorresteijn L. D. van Dijk E. J. (2015). Poststroke epilepsy is associated with a high mortality after a stroke at young age. *Stroke* 46 2309–2311. 10.1161/STROKEAHA.115.010115 26138118

[B7] Beghi E. Giussani G. (2018). Aging and the epidemiology of epilepsy. *Neuroepidemiology* 51 216–223. 10.1159/000493484 30253417

[B8] Ben Shimon M. Lenz M. Ikenberg B. Becker D. Shavit Stein E. Chapman J. (2015). Thrombin regulation of synaptic transmission and plasticity: implications for health and disease. *Front. Cell Neurosci.* 9:151. 10.3389/fncel.2015.00151 25954157 PMC4404867

[B9] Cao L. Pokorney S. D. Hayden K. Welsh-Bohmer K. Newby L. K. (2015). Cognitive function: is there more to anticoagulation in atrial fibrillation than stroke? *J. Am. Heart Assoc*. 4:e001573. 10.1161/JAHA.114.001573 26240065 PMC4599450

[B10] Cerasa A. Fasano A. Morgante F. Koch G. Quattrone A. (2014). Maladaptive plasticity in levodopa-induced dyskinesias and tardive dyskinesias: old and new insights on the effects of dopamine receptor pharmacology. *Front. Neurol.* 5:49. 10.3389/fneur.2014.00049 24782822 PMC3988357

[B11] Chen B. Cheng Q. Yang K. Lyden P. D. (2010). Thrombin mediates severe neurovascular injury during ischemia. *Stroke* 41 2348–2352. 10.1161/STROKEAHA.110.584920 20705928

[B12] Chen B. Friedman B. Whitney M. A. Winkle J. A. Lei I. F. Olson E. S. (2012). Thrombin activity associated with neuronal damage during acute focal ischemia. *J. Neurosci.* 32 7622–7631. 10.1523/JNEUROSCI.0369-12.2012 22649241 PMC3383068

[B13] Coughlin S. R. (2000). Thrombin signalling and protease-activated receptors. *Nature* 407 258–264. 10.1038/35025229 11001069

[B14] De Pittà M. Brunel N. (2016). Modulation of synaptic plasticity by glutamatergic gliotransmission: a modeling study. *Neural Plast.* 2016 1–30. 10.1155/2016/7607924 27195153 PMC4852535

[B15] De Reuck J. (2009). Management of stroke-related seizures. *Acta Neurol. Belg.* 109 271–276. 20120206

[B16] Feher G. Gurdan Z. Gombos K. Koltai K. Pusch G. Tibold A. (2019). Early seizures after ischemic stroke: focus on thrombolysis. *CNS Spectr.* 10.1017/S1092852919000804 [Epub ahead of print]. 30915936

[B17] Feyissa A. M. Hasan T. F. Meschia J. F. (2019). Stroke-related epilepsy. *Eur. J. Neurol.* 26 18–e3. 10.1111/ene.13813 30320425

[B18] Friedman A. Kaufer D. Heinemann U. (2009). Blood-brain barrier breakdown-inducing astrocytic transformation: novel targets for the prevention of epilepsy. *Epilepsy Res*. 85 142–149. 10.1016/j.eplepsyres.2009.03.005 19362806 PMC3615244

[B19] Gilad R. (2012). Management of seizures following a stroke. *Drugs Aging* 29 533–538. 10.2165/11631540-000000000-00000 22540349

[B20] Gingrich M. B. Junge C. E. Lyuboslavsky P. Traynelis S. F. (2000). Potentiation of NMDA receptor function by the serine protease thrombin. *J. Neurosci.* 20 4582–4595. 10.1523/jneurosci.20-12-04582.2000 10844028 PMC6772448

[B21] Gingrich M. B. Traynelis S. F. (2000). Serine proteases and brain damage – is there a link? *Trends Neurosci*. 23 399–407. 10.1016/S0166-2236(00)01617-9 10941185

[B22] Göbel K. Eichler S. Wiendl H. Chavakis T. Kleinschnitz C. Meuth S. G. (2018). The coagulation factors fibrinogen, thrombin, and factor XII in inflammatory disorders—a systematic review. *Front. Immunol.* 9:1731. 10.3389/fimmu.2018.01731 30105021 PMC6077258

[B23] Graham N. S. N. Crichton S. Koutroumanidis M. Wolfe C. D. A. Rudd A. G. (2013). Incidence and associations of poststroke epilepsy. *Stroke* 44 605–611. 10.1161/STROKEAHA.111.000220 23370202

[B24] He Z. Jin Y. (2016). Intrinsic control of axon regeneration. *Neuron* 90 437–451. 10.1016/j.neuron.2016.04.022 27151637

[B25] Isaeva E. Hernan A. Isaev D. Holmes G. L. (2012). Thrombin facilitates seizures through activation of persistent sodium current. *Ann. Neurol.* 72 192–198. 10.1002/ana.23587 22926852 PMC3430976

[B26] Kim S. Y. Senatorov V. V. Morrissey C. S. Lippmann K. Vazquez O. Milikovsky D. Z. (2017). TGFβ signaling is associated with changes in inflammatory gene expression and perineuronal net degradation around inhibitory neurons following various neurological insults. *Sci. Rep.* 7:7711. 10.1038/s41598-017-07394-3 28794441 PMC5550510

[B27] Lai T. W. Zhang S. Wang Y. T. (2014). Excitotoxicity and stroke: identifying novel targets for neuroprotection. *Prog. Neurobiol.* 115 157–188. 10.1016/j.pneurobio.2013.11.006 24361499

[B28] Lamy C. Domigo V. Semah F. Arquizan C. Trystram D. Coste J. (2003). Early and late seizures after cryptogenic ischemic stroke in young adults. *Neurology* 60 400–404. 10.1212/WNL.60.3.400 12578918

[B29] Lee K. R. Drury I. Vitarbo E. Hoff J. T. (1997). Seizures induced by intracerebral injection of thrombin: a model of intracerebral hemorrhage. *J. Neurosurg.* 87 73–78. 10.3171/jns.1997.87.1.0073 9202268

[B30] Lenz M. Vlachos A. Maggio N. (2015). Ischemic long-term-potentiation (iLTP), perspectives to set the threshold of neural plasticity toward therapy. *Neural Regen. Res.* 10 1537–1539. 10.4103/1673-5374.165215 26692832 PMC4660728

[B31] Leone M. A. Tonini M. C. Bogliun G. Gionco M. Tassinari T. Bottacchi E. (2009). Risk factors for a first epileptic seizure after stroke: a case control study. *J. Neurol. Sci.* 277 138–142. 10.1016/j.jns.2008.11.004 19059614

[B32] Luscher C. Malenka R. C. (2012). NMDA Receptor-Dependent Long-term potentiation and long-term depression (LTP/LTD). *Cold Spring Harb. Perspect. Biol.* 4:a005710. 10.1101/cshperspect.a005710 22510460 PMC3367554

[B33] Lyubkin M. Durand D. M. Haxhiu M. A. (1997). Interaction between tetanus long-term potentiation and hypoxia-induced potentiation in the rat hippocampus. *J. Neurophysiol.* 78 2475–2482. 10.1152/jn.1997.78.5.2475 9356398

[B34] Maggio N. Cavaliere C. Papa M. Blatt I. Chapman J. Segal M. (2013). Thrombin regulation of synaptic transmission: implications for seizure onset. *Neurobiol. Dis.* 50 171–178. 10.1016/j.nbd.2012.10.017 23103417

[B35] Mhatre M. (2004). Thrombin, a mediator of neurotoxicity and memory impairment. *Neurobiol. Aging* 25 783–793. 10.1016/S0197-4580(03)00192-1 15165703

[B36] Murphy T. R. Binder D. K. Fiacco T. A. (2017). Turning down the volume: astrocyte volume change in the generation and termination of epileptic seizures. *Neurobiol. Dis.* 104 24–32. 10.1016/j.nbd.2017.04.016 28438505 PMC5522738

[B37] Myint P. K. (2006). Post-stroke seizure and post-stroke epilepsy. *Postgrad. Med. J.* 82 568–572. 10.1136/pgmj.2005.041426 16954451 PMC2585721

[B38] Nabavi S. Fox R. Proulx C. D. Lin J. Y. Tsien R. Y. Malinow R. (2014). Engineering a memory with LTD and LTP. *Nature* 511 348–352. 10.1038/nature13294 24896183 PMC4210354

[B39] Noorbakhsh F. Vergnolle N. Hollenberg M. D. Power C. (2003). Proteinase-activated receptors in the nervous system. *Nat. Rev. Neurosci.* 4 981–990. 10.1038/nrn1255 14682360

[B40] Orfila J. E. McKinnon N. Moreno M. Deng G. Chalmers N. Dietz R. M. (2018). Cardiac arrest induces ischemic long-term potentiation of hippocampal CA1 neurons that occludes physiological long-term potentiation. *Neural Plast.* 2018:9275239. 10.1155/2018/9275239 29853851 PMC5944194

[B41] Park H. Han K.-S. Seo J. Lee J. Dravid S. M. Woo J. (2015). Channel-mediated astrocytic glutamate modulates hippocampal synaptic plasticity by activating postsynaptic NMDA receptors. *Mol. Brain* 8:7. 10.1186/s13041-015-0097-y 25645137 PMC4320468

[B42] Pitkänen A. Roivainen R. Lukasiuk K. (2016). Development of epilepsy after ischaemic stroke. *Lancet Neurol.* 15 185–197. 10.1016/S1474-4422(15)00248-3 26597090

[B43] Pleşeru A.-M. Mihailă R. G. (2018). The role of thrombin in central nervous system activity and stroke. *Clujul Med.* 91 368–371. 10.15386/cjmed-973 30564010 PMC6296729

[B44] Rumpel S. (2005). Postsynaptic receptor trafficking underlying a form of associative learning. *Science* 308 83–88. 10.1126/science.1103944 15746389

[B45] Sarecka-Hujar B. Kopyta I. (2019). Poststroke epilepsy: current perspectives on diagnosis and treatment. *Neuropsychiatr. Dis. Treat.* 15 95–103. 10.2147/NDT.S169579 30636875 PMC6309019

[B46] Schuldt G. Galanis C. Strehl A. Hick M. Schiener S. Lenz M. (2016). Inhibition of protease-activated receptor 1 does not affect dendritic homeostasis of cultured mouse dentate granule cells. *Front. Neuroanat.* 10:64. 10.3389/fnana.2016.00064 27378862 PMC4904007

[B47] Silverman I. E. Restrepo L. Mathews G. C. (2002). Poststroke Seizures. *Arch. Neurol.* 59:195. 10.1001/archneur.59.2.195 11843689

[B48] Stefanidou M. Das R. R. Beiser A. S. Sundar B. Kelly-Hayes M. Kase C. S. (2017). Incidence of seizures following initial ischemic stroke in a community-based cohort: the framingham heart study. *Seizure* 47 105–110. 10.1016/j.seizure.2017.03.009 28364691

[B49] Stein E. S. Itsekson-Hayosh Z. Aronovich A. Reisner Y. Bushi D. Pick C. G. (2015). Thrombin induces ischemic LTP (iLTP), implications for synaptic plasticity in the acute phase of ischemic stroke. *Sci. Rep.* 5:7912. 10.1038/srep07912 25604482 PMC4300504

[B50] Szaflarski J. P. Rackley A. Y. Kleindorfer D. O. Khoury J. Woo D. Miller R. (2008). Incidence of seizures in the acute phase of stroke: a population-based study HHS Public Access. *Epilepsia* 49 974–981. 10.1111/j.1528-1167.2007.01513.x 18248443 PMC5316476

[B51] Terunuma M. Xu J. Vithlani M. Pangalos M. Pg H. Da C. (2019). Current Literature in Basic Science Impact of Protein Kinase C Activation on Status Epilepticus And Epileptogenesis: Oh, What A Tangled Web Deficits in Phosphorylation of GABA A Receptors by Intimately Associated Protein Kinase C Activity Underlie Com-Promised Synaptic Inhibition during Status Epilepticus. Available at: https://www.ncbi.nlm.nih.gov/pmc/articles/PMC2442157/pdf/epc0008-0110.pdf (accessed May 13, 2019).

[B52] Tomari S. Tanaka T. Ihara M. Matsuki T. Fukuma K. Matsubara S. (2017). Risk factors for post-stroke seizure recurrence after the first episode. *Seizure* 52 22–26. 10.1016/j.seizure.2017.09.007 28957721

[B53] Wang J. Z. Vyas M. V. Saposnik G. Burneo J. G. (2017). Incidence and management of seizures after ischemic stroke. *Neurology* 89 1220–1228. 10.1212/WNL.0000000000004407 28835405

[B54] Xu M. Y. (2019). Poststroke seizure: optimising its management. *Stroke Vasc. Neurol.* 4 48–56. 10.1136/svn-2018-000175 31105979 PMC6475084

[B55] Yang C. Hawkins K. E. Doré S. Candelario-Jalil E. (2019). Neuroinflammatory mechanisms of blood-brain barrier damage in ischemic stroke. *Am. J. Physiol. Physiol.* 316 C135–C153. 10.1152/ajpcell.00136.2018 30379577 PMC6397344

[B56] Zhao Y. Li X. Zhang K. Tong T. Cui R. (2018). The progress of epilepsy after stroke. *Curr. Neuropharmacol.* 16 71–78. 10.2174/1570159X15666170613083253 28606039 PMC5771387

[B57] Zhou H.-H. Tang Y. Zhang X.-Y. Luo C. X. Gao L. Y. Wu H. Y. (2015). Delayed administration of Tat-HA-NR2B9c promotes recovery after stroke in rats. *Stroke* 46 1352–1358. 10.1161/STROKEAHA.115.008886 10.1161/STROKEAHA.115.008886 25851770

